# A systematic analysis of affinity tags in the haloarchaeal expression system, *Haloferax volcanii* for protein purification

**DOI:** 10.3389/fmicb.2024.1403623

**Published:** 2024-05-30

**Authors:** Ram Karan, Dominik Renn, Thorsten Allers, Magnus Rueping

**Affiliations:** ^1^Department of Microbiology, University of Delhi, South Campus, New Delhi, India; ^2^King Abdullah University of Science and Technology (KAUST), KAUST Catalysis Center, Thuwal, Makkah, Saudi Arabia; ^3^School of Life Sciences, University of Nottingham, Queen’s Medical Centre, Nottingham, United Kingdom; ^4^Institute for Experimental Molecular Imaging, University Clinic, RWTH Aachen University, Aachen, Germany

**Keywords:** halophiles, archaea, extremophiles, purification tags, His-tag, Strep-tag^®^II, *Haloferax volcanii*, bioengineering

## Abstract

Extremophilic proteins are valuable in various fields, but their expression can be challenging in traditional hosts like *Escherichia coli* due to misfolding and aggregation. *Haloferax volcanii* (*H. volcanii*), a halophilic expression system, offers a solution. This study examined cleavable and non-cleavable purification tags at both the N- and C-termini when fused with the superfolder green fluorescent protein (sfGFP) in *H. volcanii*. Our findings reveal that an N-terminal 8xHis-tag or Strep-tag^®^II significantly enhances protein production, purity, and yield in *H. volcanii*. Further experiments with mCherry and halophilic alcohol dehydrogenase (ADH) showed improved expression and purification yields when the 8xHis-tag or Strep-tag^®^II was positioned at the C-terminus for mCherry and at the N-terminus for ADH. Co-positioning 8xHis-tag and Twin-Strep-tag^®^ at the N-terminus of sfGFP, mCherry, and ADH yielded significantly enhanced results. These findings highlight the importance of thoughtful purification tag design and selection in *H. volcanii*, providing valuable insights for improving protein production and purification with the potential to advance biotechnological applications.

## Graphical Abstract

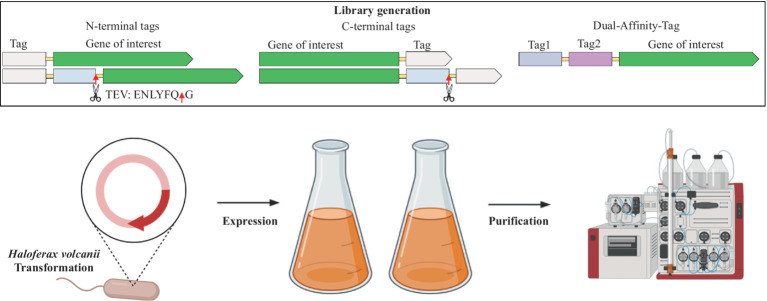

## Introduction

Extremophiles thrive in extreme physical or geochemical conditions that are inhospitable to most life forms. These environments encompass extreme temperatures, significant variations in salinity, high pressures, the presence of heavy metals, and drastic pH levels (Sysoev et al., 2021). Extremophilic proteins hold a unique position in biotechnology due to their exceptional stability under harsh environmental conditions (Wang et al., 2024). They contribute to sustainable biotechnology on Earth and offer valuable insights into the potential existence of life on other planets (Allers, 2010; Schultz et al., 2023; Wang et al., 2024). The efficient expression and cost-effective purification of recombinant proteins are paramount in today’s pharmaceutical and biotechnology industries (Puetz and Wurm, 2019). While *Escherichia coli* (*E. coli*) expression systems are widely adopted for producing heterologous recombinant proteins, they come with inherent limitations, including a limited capacity for forming disulfide bonds, the inability to perform posttranslational modifications, and the absence of an efficient secretion system (Baeshen et al., 2015; Ibrahim et al., 2023). Extremophilic proteins, which are adapted to extremes of temperature, pH, or salinity, often elude successful expression in mesophilic systems like *E. coli* due to issues of protein misfolding, conformational stress, and the formation of inclusion bodies (Elleuche et al., 2014; Kruglikov et al., 2022). Consequently, there is a pressing demand for alternative expression systems that can effectively accommodate the expression of extremophilic proteins in their native state.

*Haloferax volcanii* (*H. volcanii*) offers a solution to these challenges as an extremophilic protein expression host. *H. volcanii* is an obligate halophilic archaeon, originally isolated from the Dead Sea, which naturally thrives in extreme conditions (Strillinger et al., 2016; Grötzinger et al., 2018; Akal et al., 2019; Sysoev et al., 2021). *H. volcanii* has been equipped with a suite of microbiological and molecular genetics techniques, including an efficient DNA transformation system, shuttle plasmids, and selectable markers, making it a versatile platform for genetic studies, proteomics, and biotechnology research (Allers, 2010; Strillinger et al., 2016; Haque et al., 2019; Pérez-Arnaiz et al., 2020; Karan et al., 2023). Moreover, *H. volcanii* grows, compared to other extreme halophiles, under laboratory culture conditions at high density and in a wide range of temperatures (37–55°C) and salt concentrations (1.8–3.5 M NaCl) (Haque et al., 2019, 2020; Sysoev et al., 2021). Due to the fragile nature of the outer S-layer cell walls, *H. volcanii cells easily* lyse in hypotonic conditions, such as low salt buffer or water, releasing all cellular proteins (Dyall-Smith, 2008). This allows a simplified downstream protein purification with minimal labor and cost, making *H. volcanii* attractive for biotechnology use (Grötzinger et al., 2018; Akal et al., 2019; Karan et al., 2020, 2023; Alshehri et al., 2022).

The *H. volcanii* strain H1895, coupled with the pTA1992 plasmid, is a dedicated host with genetic modifications for enhanced protein overexpression and purification (Haque et al., 2020). Several genetic modifications have been made to *H. volcanii* strain H1895 to optimize it as an expression host for protein overexpression. Notably, biofilm-forming genes HVO_1033 and HVO_1034 have been intentionally deleted. Additionally, naturally histidine-rich proteins PitA and Cdc48d have been replaced with orthologous genes containing fewer histidine residues (Allers et al., 2010; Strillinger et al., 2016; Haque et al., 2019, 2020). These changes serve a dual purpose: they reduce potential contaminants and enable polyhistidine-tag (His-tag) purification, a common method in protein purification. Moreover, H1895 has proven to be an excellent platform for expressing various enzymes and larger molecular structures (Allers, 2010; Strillinger et al., 2016; Haque et al., 2019, 2020; Karan et al., 2023). Furthermore, the lack of biofilm formation extends its utility to bioreactor applications, further underlining its versatility and potential for biotechnological endeavors (Strillinger et al., 2016; Haque et al., 2020).

Functional protein expression and purification are essential for exploring protein structure, function, and biotechnological applications (Du et al., 2022). Therefore, applying appropriate purification tags that enhance solubility and are cleavable in protein production is crucial. These tags help achieve high expression yields, target protein purity, save time, and reduce production costs (Gomari et al., 2020). However, it is important to note that the construction of fusion proteins can often affect the expression level, solubility, stability, and subsequent purification efficiency of recombinant proteins (Yadav et al., 2016; Köppl et al., 2022; Le et al., 2022). Even in well-studied expression systems like *E. coli*, there is no universal rule for determining the optimal expression conditions, purification tag types, and positions (Köppl et al., 2022). Regarding haloarchaeal proteins, expression levels are typically low, and the procedures for purifying these proteins can be complex and costly (Martínez-Espinosa, 2019). Systematic studies are missing to investigate the effects of purification tag positions and the influence of commonly used affinity and solubility-enhancing tags on expression, solubility, and purification in haloarchaeal expression systems.

This study systematically investigates the impact of various purification tags on protein expression, solubility, and subsequent purification of *H. volcanii* expressed proteins. Consequently, fusion constructs using the superfolder green fluorescent protein (sfGFP) and a range of common purification tags (polyhistidine-tag or His-tag, Strep-tag^®^II, Twin-Strep-tag^®^, a FLAG-tag, and 3x-FLAG-tag), both at the N- and C-termini, with and without cleavage sites were designed (Skerra and Schmidt, 1999; Einhauer and Jungbauer, 2001; Kimple and Sondek, 2004; Pédelacq et al., 2006; Shahravan et al., 2008; Allers et al., 2010; Hermans et al., 2012; Schmidt et al., 2012, 2013; Young et al., 2012; Beznosov et al., 2013; Kimple et al., 2013; Zhao et al., 2013; Djender et al., 2014; Nguyen et al., 2014; Yeliseev et al., 2017; Mishra, 2020; Köppl et al., 2022). Next, the study also investigates the novel four amino acid short C-tag at the C-terminus of sfGFP to diversify the tag library further. Fluorescence measurements of sfGFP enable rapid and efficient expression, solubility, and purification estimations of these fusion proteins (Pédelacq et al., 2006). Furthermore, a comparison was conducted using 8xHis-tag and Strep-tag^®^II at both the N- and C-termini with the red fluorescent protein mCherry and a previously fully characterized halophilic alcohol dehydrogenase (ADH) from the deep-sea brine pool of the Red Sea (Shaner et al., 2004; Grötzinger et al., 2018; Akal et al., 2019). Additionally, the dual-affinity-tag approach by combining the 8xHis-tag and the Strep-tag^®^II at the N-terminus of sfGFP, mCherry, and ADH was explored. These investigations unveiled the substantial impact of different tags, tag lengths, and positions on protein expression and purification. The insights gained from this study hold significant promise in addressing the challenges associated with producing and purifying halophilic proteins in *H. volcanii*.

## Materials and methods

### Materials

All chemicals and solvents were purchased from Sigma-Aldrich (St. Louis, MO, United States). Water was desalted and purified using a milli-Q^®^ (Merck, Darmstadt, Germany) system. This study used *Haloferax volcanii* H1895 and the vector pTA1992 (Haque et al., 2019).

### Plasmid preparation

The purification-tag-fusion-sfGFP protein synthetic gene library (GenScript Biotech Corporation, HK), together with the mCherry and ADH constructs were codon-optimized using the java codon adaptation online tool Jcat for *Halobacterium* sp. (strain NRC-1/ATCC 700922/JCM 11081) (Grote et al., 2005) and cloned into the pTA1921 vector via NdeI and BamHI restriction enzymes (Supplementary Figure S1 and Supplementary Table S1).

### Protein expression in *Haloferax volcanii*

The construct containing vectors was transformed into the *Haloferax volcanii* H1895 using the PEG/EDTA method (Dyall-Smith, 2008). *H. volcanii* and derivatives were cultured in the Hv-YPC medium at 45°C with shaking for 24–36 h, as previously described (Strillinger et al., 2016; Grötzinger et al., 2018; Akal et al., 2019; Karan et al., 2020; Alshehri et al., 2022). For solid media, 2% (w/v) agar was added. Stock cultures were maintained in glycerol at −80°C. For short-term use, cultures were maintained on stock plates.

### Estimation of expression level and solubility

The expression level of the sfGFP or mCherry constructs was evaluated by measuring the fluorescence (ex 485 nm/em 507 nm) or mCherry (ex 587 nm/em 610 nm) of 24 h grown cell culture/OD_600_ with three different colonies. The expression level of the ADH constructs was evaluated by determining the final protein concentration after purification using NanoDrop absorption at 280 nm. The results were transformed into relative expression, with the highest fluorescence (for sfGFP N-ter Strep-tag, mCherry C-ter 8xHis-tag, and ADH N-ter 8xHis-tag) set to 100%.

The solubility of sfGFP and mCherry was evaluated by comparing the fluorescence after sonication, before and after centrifugation. For ADH, which lacks fluorescence, solubility was evaluated using SDS-PAGE and Western blotting to compare the soluble fraction against the pellet.

### Cell harvesting and lysis

One liter of cell culture was grown until an OD_600_ of 1.0–1.5. Cells were harvested by centrifugation (4,000 × g, 4°C, 45 min) in a Legend XFR Centrifuge (Thermo Scientific, Waltham, United States) and disrupted in binding buffer (50 mM sodium phosphate buffer, pH 7.4 containing 200 mM or 2 M NaCl) containing Pierce^™^ Protease Inhibitor EDTA-free tablet (Thermo Scientific) using a sonicator (Model Q500, QSONICA, Newtown, CT, United States) with a 1.9 cm probe. Cell debris was removed by centrifugation (24,000 × g, 4°C, 45 min) in a Multifuge X1R Centrifuge (Thermo Scientific).

### Purification of constructs

All constructs were purified using the ÄKTAprime plus chromatography system (GE Healthcare Life Sciences, Piscataway, NJ, United States) according to the purification tag in the binding buffer (50 mM sodium phosphate, pH 7.4, 200 mM or 2 M NaCl).

### Purification of His-tag fused constructs

The polyHis and SUMO fused constructs were purified on a 1 mL HiTrap Ni^2+^ chelating column (GE Healthcare Life Sciences, Piscataway, NJ, United States). The supernatant was loaded at a 1.0 mL/min flow rate onto the column pre-equilibrated with a binding buffer (50 mM sodium phosphate, pH 7.4, 200 mM, or 2 M NaCl) containing 20 mM imidazole. After washing the column with binding buffer, the protein was eluted in one step with His-elution buffer (binding buffer containing 250 mM imidazole).

### Purification of Strep-tag^®^II fused constructs

The Strep-tag^®^II and Twin-Strep-tag^®^ tag fused constructs were purified on a 1 mL Strep-Tactin^®^XT 4Flow (GenScript Biotech Corporation, HK). The supernatant was loaded at a 1.0 mL/min flow rate onto the column pre-equilibrated with binding buffer (50 mM sodium phosphate, pH 7.4, 200 mM, or 2 M NaCl). After washing the column with binding buffer, the protein was eluted in one step with a strep-elution buffer (binding buffer containing 50 mM biotin).

### Purification of FLAG-tag fused constructs

The FLAG-tag and 3X FLAG-tag fused constructs were purified on a 1 mL Anti-DYKDDDDK G1 Affinity Resin column (GenScript Biotech Corporation, HK). The supernatant was loaded at a 1.0 mL/min flow rate onto the column pre-equilibrated with binding buffer (50 mM sodium phosphate, pH 7.4, 200 mM, or 2 M NaCl). After washing the column with binding buffer, the protein was eluted in a one-step FLAG-elution buffer (100 mM Tris/HCl buffer, pH 12.0, 200 mM, or 2 M NaCl). To purify the FLAG/3xFLAG-tag fused constructs, MonoRab^™^ Anti-DYKDDDDK Magnetic Beads (Cat No. L00835, GenScript) were tested as well. The cell lysate was added to the pre-washed beads with bead binding buffer (50 mM sodium phosphate, pH 7.4, 200 mM or 2 M NaCl) and incubated on a shaker at 4°C for 2 h. After magnetic separation, the supernatant was removed, and the magnetic beads were washed with the bead binding buffer three times. Finally, the DYKDDDDK fused construct bound to the beads was eluted by bead elution buffer (100 mM Tris/HCl buffer, pH 12.0).

### Purification of C-tag fused constructs

The C-tag fused constructs were purified on a 1 mL CaptureSelect^™^ C-tagXL column (Thermo Scientific, Waltham, United States). The supernatant was loaded at a 1.0 mL/min flow rate onto the column pre-equilibrated with binding buffer (50 mM sodium phosphate, pH 7.4, 200 mM, or 2 M NaCl). The protein was eluted in one step with a C-tag-elution buffer (50 mM sodium phosphate, pH 7.4, 2 M MgCl_2_).

### Supplementary steps for purification and analysis

The purified fractions were combined, further purified, and concentrated with Amicon^®^ Ultra-4 Centrifugal Filter Units, 10 kDa. The protein was then dialyzed against dialysis buffer (20 mM sodium phosphate, pH 7.4, 200 mM, or 2 M NaCl). Protein concentration was determined using NanoDrop absorption at 280 nm.

Note: The purification procedures of each construct were not optimized (e.g., elution gradient, wash steps) to compare better the purity of sfGFP, respectively, mCherry and ADH, constructs for each purification tag.

### Protein cleavage

For fusion constructs containing cleavable tags, the cleavage of the purification tags was performed overnight at 4°C in dialysis buffer (50 mM Tris-HCl, 1 mM DTT, pH 8.0) containing 200 mM NaCl or 2 M NaCl, with the respective SUMO-Protease, containing a His-tag, (ratio of 1:10 w/w) and TEV-Protease, containing a His-tag, (200 U for 1 mg of fusion protein), and then reapplied to the respective affinity column to remove the cleaved purification tag. The SUMO- and TEV-Protease were removed by the HiTrap Ni^2+^ chelating column (GE Healthcare Life Sciences, Piscataway, NJ, United States).

The FLAG tag contains an internal enterokinase (EK) cleavage site, which allows the removal of the affinity tag after purification. The eluted fractions were incubated with Enterokinase, containing a His-tag (Z03376-1, Genscript, 200 U for 1 mg of fusion protein) to remove the FLAG-tag. The Enterokinase was removed by the HiTrap Ni^2+^ chelating column (GE Healthcare Life Sciences, Piscataway, NJ, United States).

### SDS-PAGE and western blotting analysis

Western blotting analysis was performed as previously described (DasSarma et al., 2013; Andar et al., 2017; Karan et al., 2020). The SDS-PAGE analysis was performed using Novex^®^ Tris-glycine gels (4–20%, Invitrogen, Carlsbad, CA, United States). Proteins were then transferred to 0.45 μm nitrocellulose membranes (Millipore Corp., Boston, MA). Membranes were blocked for 15 min in Pierce Fast Blocking Buffer (Thermo Fisher Scientific) and then incubated in Anti-GFP Polyclonal Antibody (Thermo Fisher Scientific) at 1:2000 dilution, followed by three washing steps with TBST, then incubated with Goat anti-Rabbit IgG (H + L) Cross-Adsorbed Secondary Antibody, Alexa Fluor 488 (Invitrogen, Catalog # A-11008).

The purity of the proteins was evaluated using SDS-PAGE stained with Coomassie brilliant blue and quantified with ImageJ software^1^ (Schneider et al., 2012). Purity was specified as a percentage of the total protein content, with the intensity of the target protein band compared to the total protein intensity on each lane of the gel.

### Tryptic digest and LC-MS/MS analysis

The identification of corresponding peptides was performed by LC-MS/MS analysis. 10 μg of the sample was digested with trypsin using the FASP protocol (Wiśniewski et al., 2009). Peptides were measured using an LTQ-Orbitrap mass spectrometer (Thermo Fisher Scientific, Waltham, MA, United States) and analyzed by MASCOT v2.3 (Matrix Sciences Ltd., United Kingdom).

## Results and discussion

### Experimental design for systematic screening

**Figure 1:**
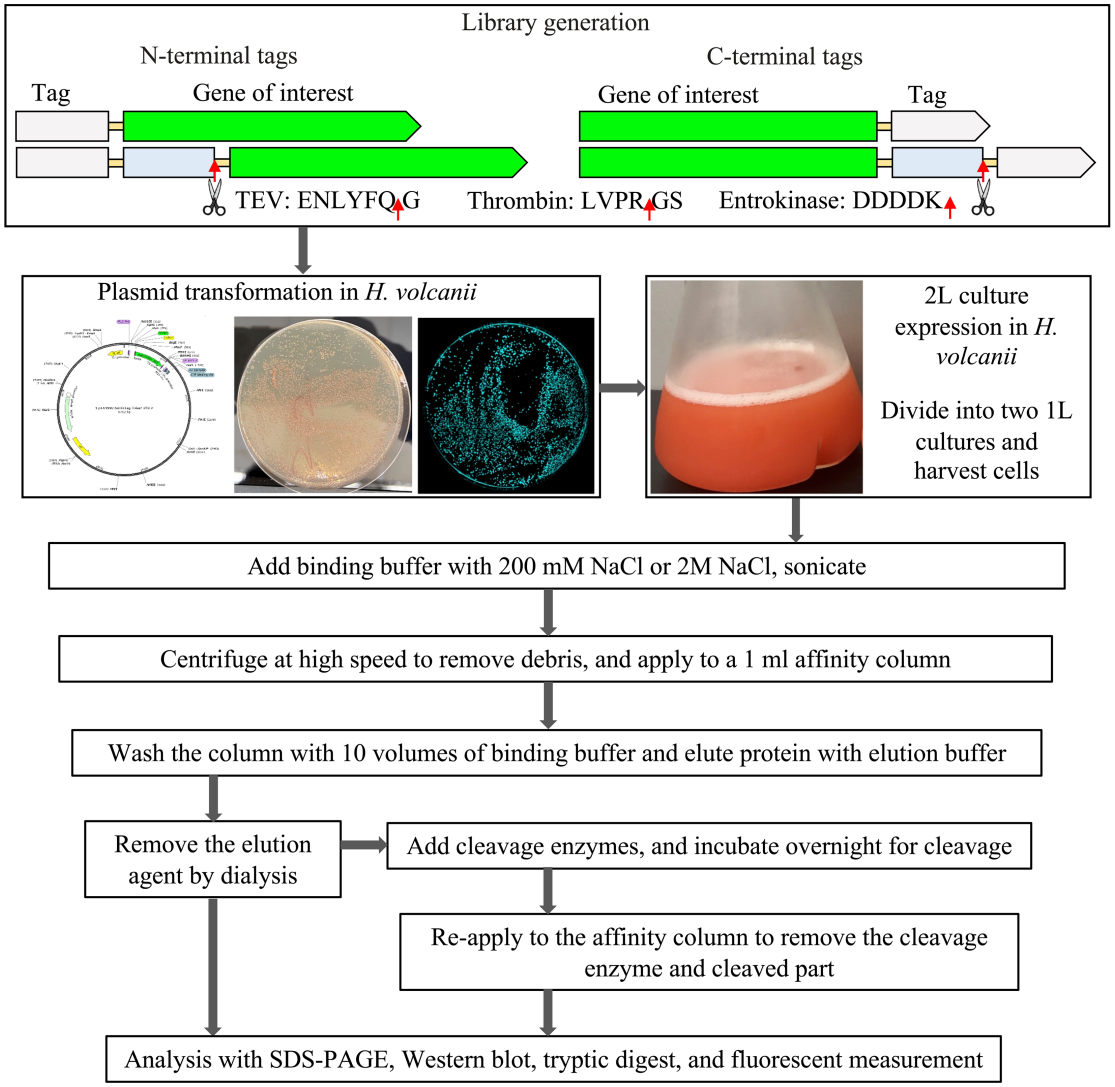
Schematic representation of constructs and purification process.

In the study, *H. volcanii* strain H1895 as the host, in combination with the pTA1992 plasmid that features the exceptionally strong constitutive p.syn promoter (Haque et al., 2019, 2020) was employed. Next, a versatile purification-tag-fusion-protein library using the sfGFP protein as a reporter (Pédelacq et al., 2006), was designed and constructed. Within this library, a range of purification tags, including 6xHis, 8xHis, Strep-tag^®^II, Twin-Strep-tag^®^, FLAG, and 3x-FLAG, strategically placed at both the N-terminus and C-terminus of sfGFP (Figure 1) were introduced.

His-tags are the most used and cost-effective affinity tags to purify recombinant proteins using an immobilized metal affinity chromatography (IMAC) column (Porath et al., 1975). Notably, protein activity is rarely affected by polyhistidine affinity tags because of their relatively small size and charge (Block et al., 2009). In the context of *H. volcanii* strains where naturally histidine-rich proteins PitA and Cdc48d have been replaced with orthologous genes containing fewer histidine residue, the 6xHis-tag has been widely adopted for recombinant protein purification (Grötzinger et al., 2018; Haque et al., 2019, 2020; Karan et al., 2020). The Small Ubiquitin-like Modifier (SUMO)-tag, which incorporated either a 6xHis-tag or an 8xHis-tag for expression and purification (Butt et al., 2005) was used for the library design. Strep-tag^®^II, an 8-amino acid peptide tag (WSHPQFEK), exhibits a strong affinity for Strep-Tactin^®^ (or Strep-Tactin^®^XT), a specially engineered streptavidin under physiological buffer conditions (Skerra and Schmidt, 1999; Schmidt et al., 2013; Yeliseev et al., 2017). This interaction allows for the rapid one-step purification of nearly any recombinant protein at high purity and functionality. Moreover, the Strep-tag^®^II can be found as a repetitive motif, generating a Twin-Strep-tag^®^. The Twin-Strep-tag^®^, characterized by its relatively higher affinity for Strep-Tactin^®^ matrices, offers enhanced purification capabilities for recombinant proteins.

The FLAG-tag is a small, highly soluble tag consisting of a hydrophilic octapeptide epitope (DYKDDDDK) with an enterokinase-cleavage site (DDDDK). This tag’s hydrophilic nature positions it primarily on the surface of the recombinant protein, increasing accessibility to resins and minimizing potential adverse effects on the fusion protein (Hopp et al., 1988; Schmidt et al., 2012). Similarly, the 3xFLAG-tag is a robust epitope tag (DYKDHDG-DYKDHDI-DYKDDDDK) was fused with sfGFP at the N- and C-termini.

A new class of purification tags, the four-peptide (E-P-E-A) short C-tag, which exhibits a strong affinity for a camelid antibody fragment when expressed at the C-terminus of a protein was also explored (Djender et al., 2014).

We also scrutinized the effect of positioning the TEV cleavage site at the N-terminus and C-terminus of the sfGFP fusion constructs (Supplementary Table S1 and Supplementary Figure S1). A total of 26 distinct sfGFP constructs were studied, each evaluated for expression through fluorescence measurements (Supplementary Figure S2), further corroborated by Western blot analysis (Supplementary Figure S3). The different sfGFP constructs were compared for their relative expression level, solubility, and purification profile (Supplementary Figure S4). Tryptic digest analysis was additionally performed to confirm the identity of the fusion proteins (Supplementary Figure S5). The focus was primarily on purification at low salt (200 mM NaCl) and high salt concentrations (2 M NaCl). This comprehensive evaluation aimed to determine the most suitable purification tag for the native purification of extremophilic halophilic proteins, capitalizing on the suitability of *H. volcanii* as the expression host.

The initial systematic screening library was expanded to examine the applicability of the sfGFP results to the red fluorescent protein mCherry (Shaner et al., 2004) and a previously fully characterized halophilic alcohol dehydrogenase (ADH) from the deep-sea brine pool of the Red Sea (Grötzinger et al., 2018) (Supplementary Figure S7). These investigations revealed the significant impact of different tags, tag lengths, and positions on protein expression and purification. This resulted in a total of 37 screened constructs.

### Expression and purification of sfGFP fusion proteins

The corresponding overview of the expression and purification of the sfGFP constructs is presented in the order of the different studied affinity purification tag systems.

### Polyhistidine-tag (His-tag)

The experimental design explored the efficacy of both a 6xHis-tag and an 8xHis-tag at the N- and C-termini of sfGFP, both with and without a TEV cleavage site. Furthermore, the N-terminal solubility-enhancing and cleavable Small Ubiquitin-like Modifier (SUMO)-tag, which incorporated either a 6xHis-tag or an 8xHis-tag for expression and purification (Butt et al., 2005), was used. All the constructs underwent purification and cleavage, where applicable, under varying salt concentrations, ranging from low (200 mM NaCl) to high (2 M NaCl) in a 50 mM sodium phosphate buffer, pH 7.4. The expression level, solubility, purity, and final yield of the fusion sfGFP constructs are presented in Table 1 and Figure 1.

**Table 1:** Relative expression, solubility, purity, and yield of sfGFP expressed with His-tag constructs.

Construct	Expression level (%)	Solubility (%)		Purity (%)		Yield (mg/L of culture)	
		Low salt	High salt	Low salt	High salt	Low salt	High salt
N-terminal purification His-tag/TEV cleavage site/ SUMO-tag							
1. 6xHis-sfGFP	51	97	94	97	75	2.4	2.8
2. 8xHis-sfGFP	95	98	93	90	85	4.9	3.5
5. 6xHis-TEV-sfGFP	49	100	92	90 (95)	85 (95)	2.5 (2.1)	2.3 (0.9)
6. 8xHis-TEV-sfGFP	75	100	96	90 (97)	85 (94)	2.8 (2.5)	1.7 (0.7)
9. 6xHis-SUMO-sfGFP	82	100	100	70 (85)	65 (80)	2.8 (2.4)	2.9 (2.1)
10. 8xHis-SUMO-sfGFP	87	100	99	65 (86)	75 (85)	3.3 (2.9)	3.6 (2.4)
C-terminal purification His-tag/TEV cleavage site							
15. sfGFP-6xHis	22	100	95	75	45	0.8	0.9
16. sfGFP-8xHis	23	100	97	70	40	0.8	1.1
20. sfGFP-TEV-6xHis	15	99	96	65 (96)	63 (90)	0.9 (0.6)	0.8 (0.4)
21. sfGFP-TEV-8xHis	17	100	97	80 (97)	85 (96)	0.8 (0.7)	0.7 (0.4)

The numbers in parentheses represent the values after cleavage and purification in the corresponding step.

### N-terminal vs. C-terminal and 6xHis- vs. 8xHis-tag

The expression of sfGFP is significantly influenced by the choice of tag and its location (Yilmaz and Arslanyolu, 2015). Notably, the highest level of sfGFP expression was achieved with the N-terminal 8xHis-tag (Table 1). Surprisingly, the introduction of the N-terminal 8xHis-SUMO-tag did not significantly enhance expression yield, and the addition of a cleavable TEV cleavage site had a noticeable impact on expression levels. Conversely, sfGFP expression with C-terminal His-tags demonstrated significantly lower levels, up to one-fifth, compared to the N-terminal 8xHis-tag. While the expression level of sfGFP varied regarding the position and length of the tag, it’s noteworthy that these variations did not substantially impact solubility. All constructs exhibited consistently high solubility levels ranging from 92 to 100%, irrespective of salt concentration. The purity of the sfGFP fusion proteins displayed notable variation concerning His-tag length, position, and salt concentration in the purification buffer. The 8xHis-tag consistently outperformed all other His-tag constructs, resulting in high purity and yield in high and low salt buffers. This outcome aligns with expectations, as the increased number of histidine residues in the tag leads to more efficient binding on IMAC columns. Furthermore, it allows for more rigorous washes, enhancing the overall purity of the protein.

### Sumo-tag vs. TEV cleavage site and the impact of salt

**Figure 2:**
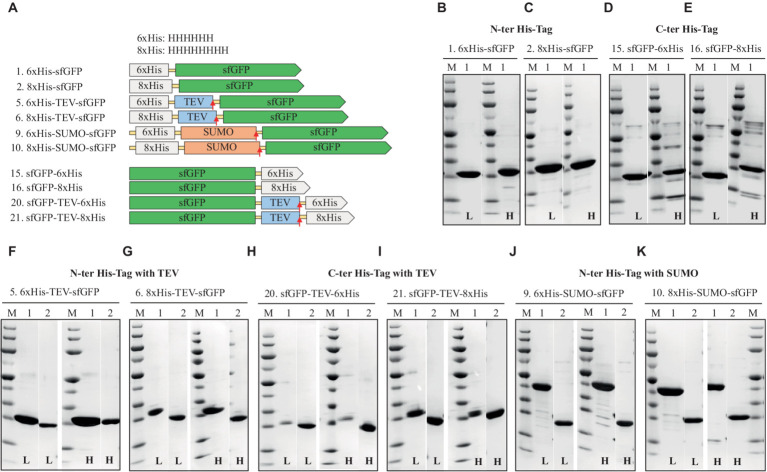
Schematic representation of His-tag constructs **(A)** and SDS-PAGE analysis of the purification of the sfGFP with N-terminal 6xHis **(B)**, 8xHis **(C)**, and C-terminal 6xHis **(D)** 8xHis **(E)**, N-terminal His-tag with cleavable tag, TEV, 6xHis, **(F)** 8xHis **(G)**, and C-terminal His-tag with TEV cleavage site, 6xHis **(H)**, 8xHis **(I)**, N-terminal His-tag with solubility and cleavable SUMO-tag with 6xHis **(J)**, or 8xHis **(K)**. Lane M: molecular weight marker; Lane 1: Elution from HisTrap affinity column using the binding buffer containing 250 mM imidazole; Lane 2: Flow-through from HisTrap affinity column loaded after TEV-/SUMO-Protease cleavage. L: Low salt (200 mM NaCl); H: High salt (2 M NaCl) in the binding, washing, and elution buffers.

A comparative assessment of the solubility-enhancing and cleavable SUMO-tag versus the TEV cleavage site yielded valuable insights into the performance of these constructs. The SUMO-tag exhibited robust performance, outperforming the cleavable TEV cleavage site. The SUMO-tag was pivotal in enhancing sfGFP expression and ensuring high purity, irrespective of the buffering composition. sfGFP constructs with the TEV cleavage site also displayed strong expression, achieving even higher purity levels. However, the TEV cleavage site did not cleave efficiently at high salt concentrations (Figure 2). This limitation resulted in approximately 40–50% final yields compared to the uncleaved constructs. Of note, the sfGFP construct featuring a C-terminal 6xHis-tag with a TEV cleavage site exhibited the lowest production level among all the tested His-tagged constructs.

Overall, the N-terminal 8xHis-tag proved the best performer, offering a simple, rapid, and highly efficient one-step purification method for sfGFP. This approach employed cost-effective materials and delivered approximately double the final yield compared to the 6xHis-tag and nearly five times the yield compared to the C-terminal 6xHis- or 8xHis-tag constructs (Table 1).

The 6xHis-tag remains the most favored purification tag for *H. volcanii* due to its compatibility with high-salt media (Haque et al., 2019). However, the results, which contradict earlier studies on N-terminal His-tagged GFP that indicated adverse effects on protein production, emphasize the ongoing challenge of selecting the optimal position and length for the His-tag within various expression systems (Nguyen et al., 2014; Phan et al., 2015; Tian et al., 2019; Le et al., 2022). This study underscores the importance of making informed choices regarding His-tag design to maximize expression levels and purification efficiency.

### Strep-tag^®^II and twin-Strep-tag^®^

Next, the sfGFP constructs fused with Strep-tag^®^II and Twin-Strep-tag^®^ were investigated.

### N-terminal vs. C-terminal and Strep-tag^®^II vs. twin-Strep-tag^®^

**Figure 3:**
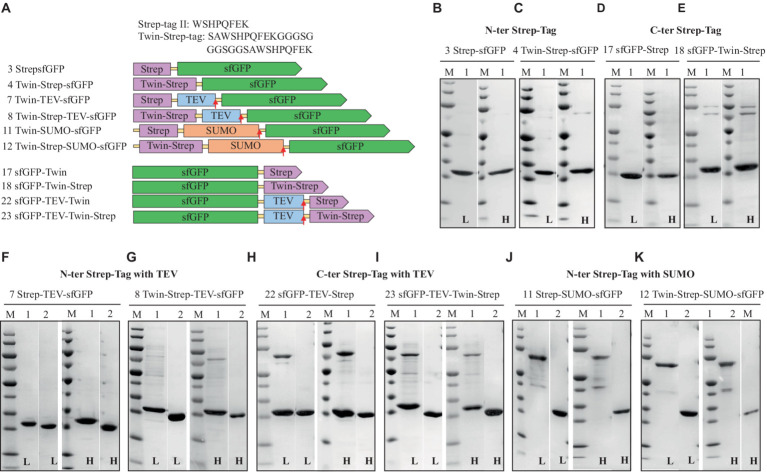
Schematic representation of Strep-tag (Strep-tag^®^II and Twin-Strep-tag^®^) constructs **(A)** and SDS-PAGE analysis of the purification of the sfGFP with N-Terminal Strep **(B)**, Twin-Strep-tag^®^ **(C)**, and C-terminal Strep **(D)** Twin-Strep-tag^®^s **(E)**. N-terminal Strep with TEV cleavage site, Strep, **(F)** Twin-Strep-tag^®^ **(G)**, and C-terminal Strep-tag^®^II with TEV cleavage site, Strep **(H)**, Twin-Strep-tag^®^s **(I)**, N-terminal Strep-tag^®^II with solubility and cleavable SUMO-Strep-tag **(J)**, and Twin-Strep-tag^®^ **(K)**. Lane M: molecular weight marker; Lane 1: Elution from Strep-Tactin affinity column using the binding buffer containing 50 mM biotin; Lane 2: Flow-through from HisTrap and Strep-Tactin affinity column loaded after TEV-/SUMO-Protease cleavage. L: Low salt (200 mM NaCl); H: High salt (2 M NaCl) in the binding, washing, and elution buffers.

The results of the sfGFP constructs fused with Strep-tag^®^II are summarized in Table 2 and Figure 3. Expression levels of sfGFP varied based on the position of the Strep-tag^®^II and Twin-Strep-tag^®^. Like the His-tag, the N-terminal Strep-tag^®^II yielded the highest sfGFP expression, surpassing the C-terminal Strep-tag^®^II or Twin-Strep-tag^®^ by up to fivefold. Notably, while C-terminal Strep-tag^®^II has been utilized for protein production in *H. volcanii* and proven compatible with high salt concentrations, the study reveals that N-terminal Strep-tag^®^II results in approximately five times higher expression, suggesting that C-terminal usage in *H. volcanii* should be reconsidered (Braun et al., 2019). Intriguingly, Twin-Strep-tag^®^, which generally exhibits a higher affinity for Strep-Tactin^®^ and greater tolerance to salts and detergents in buffers than Strep-tag^®^II (Yeliseev et al., 2017), displayed an unexpected reduction in sfGFP expression to about one-third when compared to N-terminal Strep-tag^®^II. The introduction of Strep-tags (Strep-tag^®^II or Twin-Strep-tag^®^) appeared to have no significant effect on the solubility of the fusion proteins, regardless of salt conditions. Furthermore, these tags enabled high-purity purification processes (Table 2 and Figure 2).

**Table 2:** Relative expression, solubility, and purity of sfGFP expressed with Strep-tag^®^II and Twin-Strep-tag^®^ constructs.

Construct	Expression level (%)	Solubility (%)		Purity (%)		Yield (mg/L of culture)	
		Low salt	High salt	Low salt	High salt	Low salt	High salt
N-terminal purification Strep-tag^®^II/TEV cleavage site/SUMO-tag							
3.Strep-sfGFP	100	100	85	95	75	5.3	4.3
4. Twin-Strep-sfGFP	32	100	75	84	85	2.2	1.9
7. Strep-TEV-sfGFP	27	94	88	90 (97)	87 (93)	1.9 (1.6)	2.0 (0.9)
8. Twin-Strep-TEV-sfGFP	22	100	90	79 (97)	85 (96)	1.5 (1.2)	1.3 (0.6)
11. Strep-SUMO-sfGFP	34	100	95	67 (96)	62 (95)	2.4 (1.5)	2.1 (1.2)
12. Twin-Strep-SUMO-sfGFP	27	97	100	65 (94)	71 (96)	2.3 (1.4)	2.0 (1.0)
C-terminal purification Strep-tag^®^II/TEV cleavage site							
17. sfGFP-Strep	21	91	100	90	93	1.6	1.5
18. sfGFP-Twin-Strep	17	97	94	75	70	1.7	1.9
22. sfGFP-TEV-Strep	16	100	97	70 (95)	65 (90)	1.6 (1.1)	1.7 (0.6)
23. sfGFP-TEV-Twin-Strep	15	100	100	52 (87)	64 (95)	1.9 (1.1)	1.3 (0.6)

The numbers in parentheses represent the values after cleavage and purification in the corresponding step.

### Sumo vs. TEV cleavage site and the impact of salt

Next, experiments to assess the removal of Strep-tags using protease cleavage sites (TEV and SUMO) positioned between the sfGFP protein and the Strep-tags were conducted. Subsequently, a purification step was employed to separate the sfGFP protein from the cleaved Strep-tags. Consequently, the findings indicate that both Strep-tag^®^II and Twin-Strep-tag^®^, when fused with SUMO or TEV cleavage sites, could be efficiently removed through protease cleavage (SUMO- or TEV-Protease).

Among all the tested tags fused to the N-terminus of sfGFP, Strep-tag^®^II consistently yielded the highest levels of sfGFP expression.

### Flag-tag

Next, the expression and solubility of sfGFP fused with the FLAG-tag and 3xFLAG-tag, in combination with the SUMO-tag or TEV cleavage site (Einhauer and Jungbauer, 2001; Schmidt et al., 2012; Beznosov et al., 2013) were assessed.

### N-terminal vs. C-terminal FLAG-tag and 3xFLAG-tag

Comparable to His and Strep-tagged sfGFP findings, N-terminal FLAG, and 3xFLAG-tags exhibited higher expression levels (50 and 75%, respectively) than C-terminal tags (Table 3). Interestingly, N-terminal 3xFLAG-fused sfGFP demonstrated expression levels 1.5 to 3 times superior to N-terminal FLAG-tag, C-terminal FLAG, or 3xFLAG-tagged sfGFP. Additionally, all constructs displayed high solubility, exceeding 90%, regardless of whether the FLAG or 3xFLAG was positioned at the N-terminus or C-terminus of the sfGFP protein.

**Table 3:** Relative expression, solubility, and purity of sfGFP expressed with FLAG-tag and 3xFLAG-tag.

Construct	Expression level (%)	Solubility (%)		Purity (%)		Yield (mg/L of culture)	
		Low salt	High salt	Low salt	High salt	Low salt	High salt
N-terminal purification FLAG-tag							
13. FLAG-sfGFP	50	100	92	55 (95)	92 (97)	3.6 (2.2)	2.3 (1.6)
14. 3xFLAG-sfGFP	75	100	97	90 (97)	95 (98)	3.3 (2.9)	2.9 (1.9)
C-terminal purification FLAG-tag							
25. sfGFP-FLAG	26	100	92	40	65	2.6	2.1
26. sfGFP-3xFLAG	23	97	100	55	85	2.1	1.4

The numbers in parentheses represent the values after cleavage and purification in the corresponding step.

### Cleaving the FLAG-tag/3xFLAG-tag and the impact of salt

**Figure 4:**
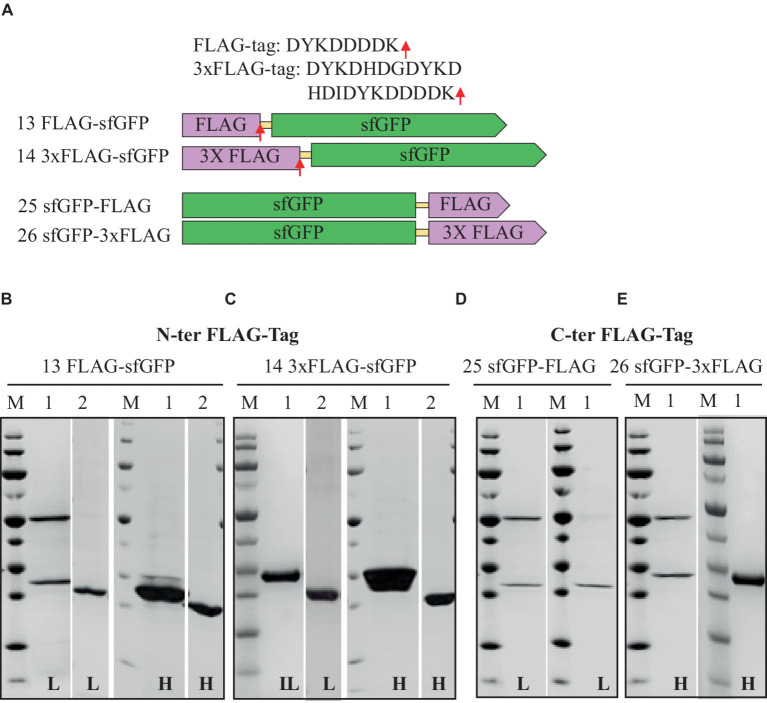
Schematic representation of FLAG-tag constructs **(A)** and SDS-PAGE analysis of the purification of the sfGFP with N-Terminal FLAG **(B)**, 3xFLAG **(C)**, and C-terminal FLAG **(D)** 3xFLAG with TEV cleavage site **(E)**. Lane M: molecular weight marker; Lane 1: Elution from anti-FLAG affinity column using 100 mM Tris/HCl buffer, pH 12.0; Lane 2: Flow-through from HisTrap affinity column loaded after TEV-Protease cleavage. L: Low salt (200 mM NaCl); H: High salt (2 M NaCl) in the binding, washing, and elution buffers.

The Enterokinase efficiently removed the FLAG-tag even at higher salt concentrations, which led to a relatively lower yield (Figure 4). All FLAG-tagged expressions resulted in highly pure sfGFP with no visible contaminating proteins on SDS-PAGE (Figure 4). To purify FLAG and 3xFLAG-tagged sfGFP from sonicated *H. volcanii* cell culture, the anti-DYKDDDDK magnetic beads (GenScript) were also tested. Remarkably, we were able to streamline the purification process by eliminating centrifugation or filtration steps and still achieved high purification (data not shown).

While FLAG-tags have previously been employed to purify recombinant proteins in various expression systems, including bacteria, baculovirus, mammalian cells, and yeast, there has been limited information regarding their use in halophilic systems (Einhauer and Jungbauer, 2001; Mishra, 2020). FLAG peptide has been tagged with modified archaellin genes (flaA1, flaA2, and flaB2) in haloarchaea *Halobacterium salinarum* cells and utilized for the identification of proteins within the archaellum (Beznosov et al., 2013). However, no reports existed on FLAG-tagged protein expression and purification in the halophilic context until this study. These findings suggest that the FLAG and 3xFLAG-tags suit protein expression and purification in halophilic systems. The FLAG-tag carries several advantages over His and Strep tags. Like the 6xHis-, 8xHis-, and Strep-tag^®^II, the FLAG-tag comprises a small hydrophilic peptide (8 amino acids), unlikely to impact protein folding or function. Furthermore, it can be easily removed with Enterokinase. However, it is worth noting that Enterokinase, while versatile, is sensitive to high salt concentrations compared to other proteases like the SUMO-Protease, limiting its application in the extremophilic environment (Shahravan et al., 2008).

### C-tag

**Figure 5:**
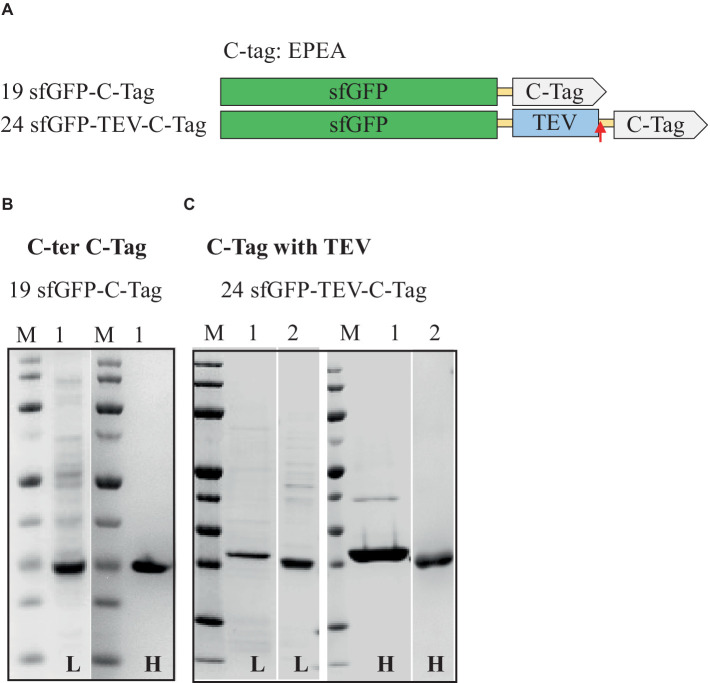
Schematic representation of C-tag constructs **(A)** and SDS-PAGE analysis of the purification of the sfGFP with C-tag **(B)** and C-tag with TEV cleavage site **(C)**. Lane M: molecular weight marker; Lane 1: Elution from C-tag XL affinity column using 2 M MgCl_2_; Lane 2: Flow-through from HisTrap affinity column loaded after TEV-Protease cleavage. L: Low salt (200 mM NaCl); H: High salt (2 M NaCl) in the binding and washing buffers.

Next, the C-tag constructs were examined, and the results detailing the expression level, solubility, purity, and final yield of the fusion sfGFP constructs are outlined in Table 4 and Figure 5. The C-tag-binding resin facilitated the purification of pure proteins in both low-salt and high-salt buffers. Notably, under the 2 M NaCl purification buffer, the protein achieved high purity at 98%, albeit with a lower yield. However, for the 200 mM NaCl purification buffer, the purity of sfGFP could be significantly improved by column washing with 1 M NaCl (Supplementary Figure S6). Moreover, including a TEV cleavage site aided in removing the C-tag, resulting in higher purity, although this came at the expense of yield, particularly in high-salt conditions due to the reduced activity of TEV-Protease in high-salt environments (Parks et al., 1995; Waugh, 2011).

**Table 4:** Relative expression, solubility, and purity of sfGFP expressed with C-tag.

Construct	Expression level (%)	Solubility (%)		Purity (%)		Yield (mg/L of culture)	
		Low salt	High salt	Low salt	High salt	Low salt	High salt
19. sfGFP-C-tag	20	100	92	92	98	0.9	0.6
24. sfGFP-TEV-C-tag	21	97	100	70 (85)	85 (95)	1.2 (0.9)	0.8 (0.4)

The numbers in parentheses represent the values after cleavage and purification in the corresponding step.

Among all the affinity tags tested, the smallest among them, the C-tag comprising just four amino acids, proved to be a rapid and efficient tool for purification. The C-tag offers several advantages over more established tags such as His, Strep, and FLAG. It is the smallest affinity purification tag with high binding affinity and selectivity. Furthermore, its limited impact on protein folding and functionality due to its small size makes it an attractive choice for applications in protein purification (Hermans et al., 2012; Djender et al., 2014; Jin et al., 2017).

The systematic screening of the different purification tags and tag positions with sfGFP revealed a significant enhancement in protein production, purity, and yield in *H. volcanii* when an 8xHis-tag or Strep-tag^®^II was placed at the N-terminus of sfGFP. This enhancement is potentially linked to favorable folding kinetics of newly synthesized proteins and increased accessibility of these tags during affinity purification, which may facilitate higher yield and purity. To explore whether this trend was unique to the expression system or exclusive to sfGFP, the investigation was extended to include the red fluorescent protein mCherry and a previously fully characterized halophilic alcohol dehydrogenase (ADH) from the deep-sea brine pool of the Red Sea (Shaner et al., 2004; Grötzinger et al., 2018).

### Transferring the expression and purification results from sfGFP to mCherry and ADH

**Figure 6:**
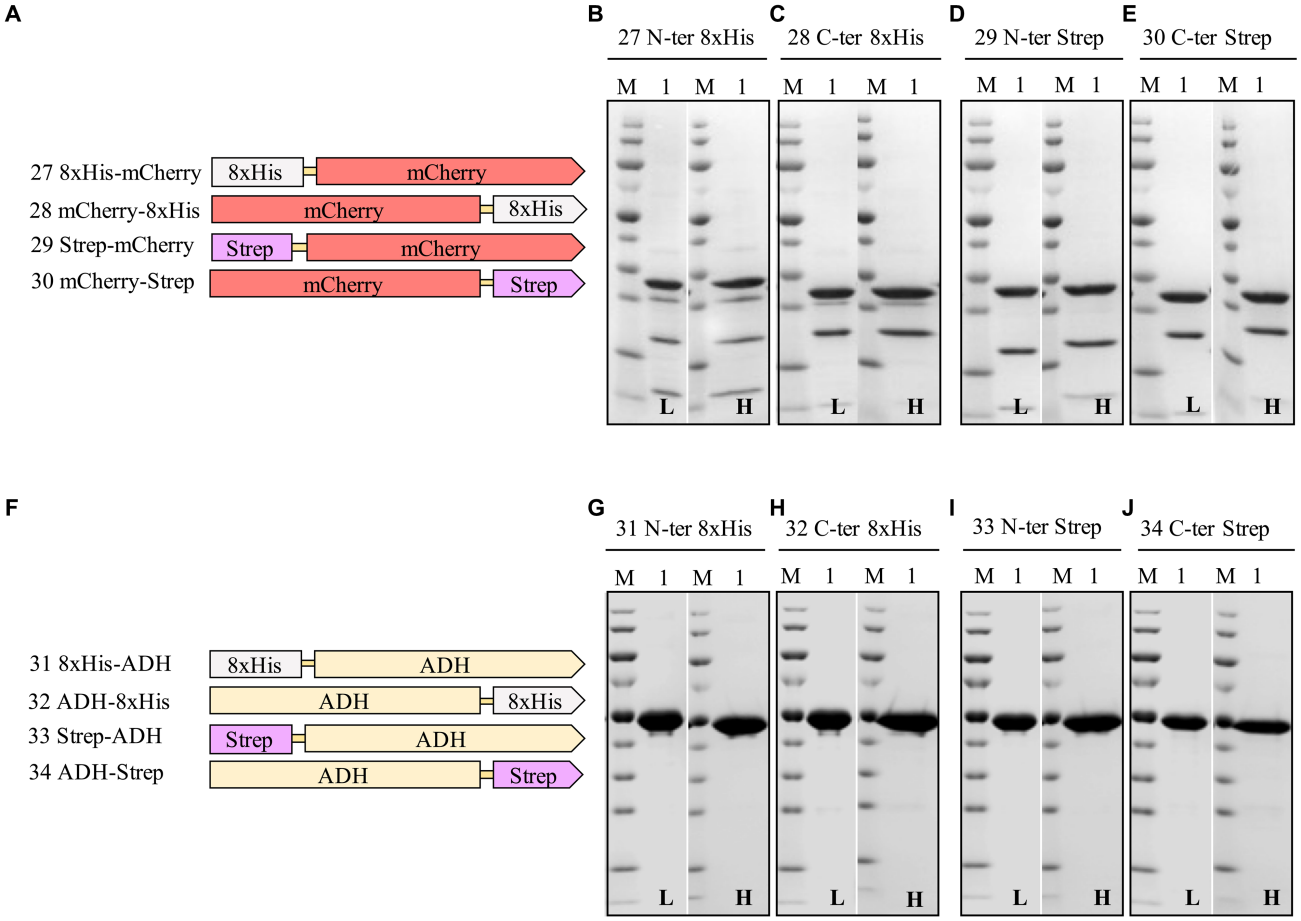
Schematic representation of N- and C-terminal 8xHis-tag and Strep-tag constructs of mCherry **(A)**, and ADH **(F)**, and SDS-PAGE analysis of the purification of the mCherry **(B–E)**, ADH **(G–J)** Lane M: molecular weight marker; Lane 1: Elution from affinity column. L: Low salt (200 mM NaCl); H: High salt (2 M NaCl) in the binding and washing buffers.

The 8xHis-tag and Strep-tag^®^II were both positioned at the N- and C-termini to investigate the impact of tag position on mCherry and the ADH (Supplementary Table S2 and Supplementary Figure S1). Surprisingly, we discovered that mCherry demonstrated improved expression levels when the 8xHis-tag, or Strep-tag^®^II, was located at the C-terminus of the protein. Conversely, ADH exhibited more efficient expression when the 8xHis-tag or Strep-tag^®^II was situated at the N-terminus of the respective proteins (Table 5 and Figure 6). Although these proteins shared similar relative solubility levels, they exhibited divergent expression patterns, emphasizing the nuanced nature of tag effects in different protein contexts.

**Table 5:** Relative expression, solubility, and purity of mCherry and ADH expressed with N- and C-terminal 8xHis-tag and Strep-tag^®^II.

Construct	Expression level^a,b^ (%)	Solubility^c^ (%)		Purity^d^ (%)		Yield^e^ (mg/L of culture)	
		Low salt	High salt	Low salt	High salt	Low salt	High salt
mCherry with 8xHis-tag							
27. 8xHis-mCherry	65	100	92	48	52	2.6	2.9
28. mCherry-8xHis	100	100	97	66	74	4.2	4.6
mCherry with Strep-tag^®^II							
29. Strep-mCherry	27	100	92	64	69	0.8	0.9
30. mCherry-Strep	96	97	100	68	75	2.4	2.8
ADH with 8xHis-tag							
31. 8xHis-ADH	100	100	92	98	99	35	34
32. ADH-8xHis	69	100	97	96	96	24	26
ADH with Strep-tag^®^II							
33. Strep-ADH	57	100	92	99	99	20	19
34. ADH-Strep	51	97	100	98	97	18	19

These findings underscore the critical role of purification tags, their positions, and lengths in determining fusion protein expression, purification, and yields. Moreover, this observation emphasizes the necessity of exploring various fusion strategies to optimize expression and purification efficiencies. We considered applying a dual-affinity-tag approach to create a more general purification tag for *H. volcanii*.

### Impact of combining 8xHis-tag and twin-Strep-tag^®^ at the N-terminus on the expression and purification of sfGFP, mCherry, and ADH: a dual-affinity-tag approach

**Figure 7:**
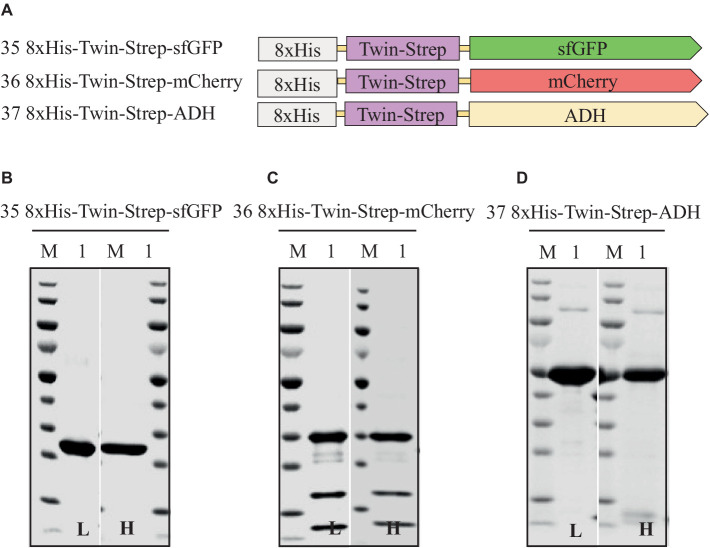
Schematic representation of N-terminal dual-affinity-tag consisting of 8xHis-tag and Twin-Strep-tag^®^ constructs of sfGFP, mCherry, and ADH **(A)** and SDS-PAGE analysis of the purification of the sfGFP **(B)**, mCherry **(C)**, ADH **(D)**. Lane M: molecular weight marker; Lane 1: Elution from affinity column. L: Low salt (200 mM NaCl); H: High salt (2 M NaCl) in the binding and washing buffers.

Assessing different tag combinations, positions, and lengths for a novel protein can be resource-intensive and time-consuming (Bernier et al., 2018). To address this challenge, we applied a dual-affinity-tag approach by combining 8xHis-tag and Twin-Strep-tag^®^ tags at the N-terminus of sfGFP, mCherry, and ADH (Supplementary Table S3 and Supplementary Figure S1). In the purification process first a HiTrap Ni^2+^ chelating column was used to exploit the 8xHis-tag, followed by a Strep-Tactin^®^XT 4Flow column to utilize the Twin-Strep-tag^®^, achieving high-purity protein. Surprisingly, this dual-affinity-tag approach consistently outperformed all previously tested tag configurations regarding expression level and purification yields (Table 6 and Figure 7). Moreover, the dual-affinity-tag seems to be a universal solution for protein expression and purification in *H. volcanii* since all tested proteins were notably expressed in higher amounts and yielded higher overall protein content.

**Table 6:** Relative expression, solubility, and purity of sfGFP, mCherry, and ADH expressed with N-terminal dual-affinity-tag consisting of 8xHis-tag and Twin-Strep-tag^®^.

Construct	Expression level^a^ (%)	Solubility^b^ (%)		Purity^c^ (%)		Yield^d^ (mg/L of culture)	
		Low salt	High salt	Low salt	High salt	Low salt	High salt
35. 8xHis-Twin-Strep-sfGFP	135	100	97	99	99	7.1	6.9
36. 8xHis-Twin-Strep-mCherry	138	100	97	57	68	5.9	5.7
37. 8xHis-Twin-Strep-ADH	109	100	98	89	86	38	42

The dual-affinity-tag approach is a method that streamlines the purification of recombinant proteins, yielding homogeneous preparations of the proteins of interest in *H. volcanii*. This is achieved by enabling two consecutive affinity chromatography steps, efficiently removing contaminating proteins.

## Conclusion

In conclusion, the study demonstrates the efficient production and purification of sfGFP proteins utilizing various affinity tags within the *Haloferax volcanii* expression system. The choice of tag and its position significantly impacts the levels of protein production and purification, with the dual-affinity-tag configuration of the 8xHis-tag and Strep-tag^®^II at the N-terminus generally providing optimal results. This approach has proven effective not just for sfGFP but also for mCherry and ADH, highlighting the importance of tag configuration over mere expression levels.

The options for cleaving the purification tag at high salt concentrations are limited due to the salt sensitivity of commonly tested proteases, including enterokinase and Tobacco Etch Virus (TEV) protease. Other frequently used proteases, such as human rhinovirus 3C protease (HRV 3C) and thrombin, also exhibit salt sensitivity (Jenny et al., 2003; Dušeková et al., 2022). However, salt-insensitive alternatives like SUMO protease and Factor Xa exist (Jenny et al., 2003). Additionally, emerging alternatives, such as the Plum Pox Virus (PPV) NIa protease, demonstrate reduced salt sensitivity, presenting viable options for such conditions (Zheng et al., 2008).

By expanding the library with mCherry and a halophilic alcohol dehydrogenase, we observed that optimal tag positioning varies depending on the protein, with C-terminal tags more effective for mCherry and N-terminal tags better suited for ADH. This underscores the nuanced approach needed in tag placement and protease selection to accommodate different protein characteristics.

As the investigation advance, incorporating proteins with inherently poor solubility will be crucial to further test and validate the adaptability of the suggested tagging strategies. This future work will enhance the general understanding of the expression system’s capabilities, providing a more comprehensive foundation for biotechnological applications in extremophilic organisms.

In summary, the study not only reinforces the significance of strategic tag design and placement but also the critical role of compatible protease selection in the successful production and purification of proteins in extremophilic systems like *Haloferax volcanii*. By focusing on these elements, researchers can optimize protein yield and purity, thereby improving the overall efficiency of their expression and purification protocols.

## Data availability statement

The original contributions presented in the study are included in the article/Supplementary material, further inquiries can be directed to the corresponding authors.

## Author contributions

RK: Conceptualization, Data curation, Formal analysis, Investigation, Methodology, Project administration, Resources, Software, Supervision, Validation, Visualization, Writing – original draft, Writing – review & editing. DR: Conceptualization, Formal analysis, Investigation, Methodology, Supervision, Validation, Writing – review & editing. TA: Resources, Validation, Writing – review & editing. MR: Conceptualization, Data curation, Formal analysis, Funding acquisition, Investigation, Methodology, Project administration, Resources, Software, Supervision, Validation, Visualization, Writing – review & editing.

## Footnotes

^1^https://imagej.nih.gov/ij/
