# Supplementary material for: A systematic analysis of affinity tags in the haloarchaeal expression system, Haloferax volcanii for protein purification
Source: Front Microbiol. 2024 May 30;15:1403623. doi: 10.3389/fmicb.2024.1403623 (PMC11169840; doi:10.3389/fmicb.2024.1403623)
Supplement: Supplementary file 1 [file Table_1.DOCX]

**SUPPLEMENTARY INFORMATION**

**A Systematic Analysis of Affinity Tags in the Haloarchaeal Expression System, *Haloferax volcanii* for Protein Purification**

Ram Karan^1,2,^*, Dominik Renn^1^*, Thorsten Allers^3^, Magnus Rueping^1,4^

^1^King Abdullah University of Science and Technology (KAUST), KAUST Catalysis Center, Thuwal 23955-6900, Saudi Arabia

^2^Department of Microbiology, University of Delhi, South Campus, New Delhi, India

^3^School of Life Sciences, University of Nottingham, Queen's Medical Centre, Nottingham, UK

^4^Institute for Experimental Molecular Imaging, University Clinic, RWTH Aachen University, Forckenbeckstrasse 55, D52074 Aachen, Germany

*Correspondence: Ram Karan [ramkaran@south.du.ac.in](mailto:ramkaran@south.du.ac.in),

Dominik Renn [dominik.renn@kaust.edu.sa](mailto:dominik.renn@kaust.edu.sa)

Table of Content

Table S1. Overview of sfGFP constructs with various purification tags used in this study. S2

Table S2. Overview of mCherry and halophilic alcohol dehydrogenase (ADH) constructs with various purification tags used in this study. S3

Table S3. Overview of sfGFP, mCherry, and halophilic alcohol dehydrogenase (ADH) constructs with N-terminal dual-affinity-tag consisting of 8xHis and Twin-Strep-tag® constructs used in this study. S3

Figure S1. Schematic representation of all the sfGFP, mCherry, and halophilic alcohol dehydrogenase (ADH) constructs with purification tags used in this study. S4

Figure S2. The expression of the sfGFP and mCherry constructs was confirmed by fluorescence imaging. S6

Figure S3. The expression of the sfGFP constructs was confirmed by Western blot analysis. S7

Figure S4. The expression and yield profile of the sfGFP, mCherry and ADH. S8

Figure S5. Tryptic digest and LC-MS/MS analysis. S9

Figure S6. SDS-PAGE analysis of the purification of the sfGFP with C-tag. S15

Figure S7. Nucleotide sequences of sfGFP, mCherry and ADH. S16

**Supplementary Table S1.** Overview of sfGFP constructs with various purification tags used in this study.

| **S.No.** | **Construct** | **Purification Tag** | **Cleavable Site** |
| --- | --- | --- | --- |
| **N-terminal purification Tags** | | | |
| 1 | 6xHis-sfGFP | 6xHis | - |
| 2 | 8xHis-sfGFP | 8xHis | - |
| 3 | Strep-sfGFP | Strep-tag®II | - |
| 4 | Twin-Strep-sfGFP | Twin-Strep-tag® | - |
| **N-terminal purification and cleavable Tags** | | | |
| 5 | 6xHis-TEV-sfGFP | 6xHis | TEV |
| 6 | 8xHis-TEV-sfGFP | 8xHis | TEV |
| 7 | Strep-TEV-sfGFP | Strep-tag®II | TEV |
| 8 | Twin-Strep-TEV-sfGFP | Twin-Strep-tag® | TEV |
| 9 | SUMO-6xHis-sfGFP | 6xHis | SUMO |
| 10 | SUMO-8xHis-sfGFP | 8xHis | SUMO |
| 11 | Strep-SUMO-sfGFP | Strep-tag®II | SUMO |
| 12 | Twin-Strep-SUMO-sfGFP | Twin-Strep-tag® | SUMO |
| 13 | FLAG-sfGFP | FLAG | Enterokinase |
| 14 | 3xFLAG-sfGFP | 3xFLAG | Enterokinase |
| **C-terminal purification Tags** | | | |
| 15 | sfGFP-6xHis | 6xHis | - |
| 16 | sfGFP-8xHis | 8xHis | - |
| 17 | sfGFP-Strep | Strep-tag®II | - |
| 18 | sfGFP-Twin-Strep | Twin-Strep-tag® | - |
| 19 | sfGFP-Ctag | C-tag | - |
| **C-terminal purification and cleavable Tags** | | | |
| 20 | sfGFP-TEV-6xHis | 6xHis | TEV |
| 21 | sfGFP-TEV-8xHis | 8xHis | TEV |
| 22 | sfGFP-TEV-Strep | Strep-tag®II | TEV |
| 23 | sfGFP-TEV-Twin-Strep | Twin-Strep-tag® | TEV |
| 24 | sfGFP-TEV-Ctag | C-tag | TEV |
| 25 | sfGFP-FLAG | FLAG | Enterokinase |
| 26 | sfGFP-3xFLAG | 3xFLAG | Enterokinase |

**Supplementary Table S2.** Overview of mCherry and halophilic alcohol dehydrogenase (ADH) constructs with various purification tags used in this study.

| **S.No.** | **Construct** | **Purification Tag and position** |
| --- | --- | --- |
| **mCherry** | | |
| 27 | 8xHis-mCherry | N-terminal 8xHis |
| 28 | mCherry-8xHis | C-terminal 8xHis |
| 29 | Strep-mCherry | N-terminal Strep-tag®II |
| 30 | mCherry-Strep | C-terminal Strep-tag®II |
| **ADH** | | |
| 31 | 8xHis-ADH | N-terminal 8xHis |
| 32 | AFD-8xHis | C-terminal 8xHis |
| 33 | Strep-ADH | N-terminal Strep-tag®II |
| 34 | ADH-Strep | C-terminal Strep-tag®II |

**Supplementary Table S3.** Overview of sfGFP, mCherry, and halophilic alcohol dehydrogenase (ADH) constructs with N-terminal dual-affinity-tag consisting of 8xHis and Twin-Strep-tag® constructs used in this study.

| **S.No.** | **Construct** | **Purification Tag and position** |
| --- | --- | --- |
| **sfGFP** | | |
| 35 | 8xHis-Twin-Strep-sfGFP | N-terminal 8xHis and Twin-Strep-tag® |
| **mCherry** | | |
| 36 | 8xHis-Twin-Strep-mCherry | N-terminal 8xHis and Twin-Strep-tag® |
| **ADH** | | |
| 37 | 8xHis-Twin-Strep-ADH | N-terminal 8xHis and Twin-Strep-tag® |

**Figure S1** Schematic representation of all the sfGFP, mCherry, and halophilic alcohol dehydrogenase (ADH) constructs with purification tags used in this study.

**Figure S2.** The expression of the sfGFP and mCherry constructs was confirmed by fluorescence imaging. Numbering corresponds to constructs described in Table S1. A fluorescent signal was detected using the iBright (ThermoFisher) imager.

**Figure S3.** The expression of the sfGFP constructs was confirmed by Western blot analysis.
Top: Cell lysate in 200 mM NaCl (low salt buffer). Bottom: Cell lysate in 2 M NaCl (high salt buffer). Numbering corresponds to constructs described in Table S1.

**Figure S4.** The expression (orange), final yield (blue for low salt and grey for high salt) of the sfGFP, mCherry and alcohol dehydrogenase (ADH). Numbering corresponds to constructs described in Table S1. The expression level of the sfGFP or mCherry constructs was evaluated by measuring the fluorescence (ex 485 nm/ em 507 nm) or mCherry (ex 587 nm/ em 610nm) of 24 h grown cell culture/OD600 with three different colonies. The expression level of the ADH constructs was evaluated by determining the final protein concentration after purification using NanoDrop absorption at 280 nm. The results were transformed into relative expression, with the highest fluorescence (for sfGFP N-ter Strep-tag, mCherry C-ter 8xHis-tag, and ADH N-ter 8xHis-tag) set to 100%.

1. **6xHis-sfGFP 1**

MHHHHHHSSGSKGEELFTGVVPILVELDGDVNGHKFSVRGEGEGDATNGKLTLKFICTTGKLPVPWPTLVTTLTYGVQCFSRYPDHMKRHDFFKSAMPEGYVQERTISFKDDGTYKTRAEVKFEGDTLVNRIELKGIDFKEDGNILGHKLEYNFNSHNVYITADKQKNGIKANFKIRHNVEDGSVQLADHYQQNTPIGDGPVLLPDNHYLSTQSVLSKDPNEKRDHMVLLEFVTAAGITHGMDELYK

1. **8xHis-sfGFP**

MHHHHHHHHSSGSKGEELFTGVVPILVELDGDVNGHKFSVRGEGEGDATNGKLTLKFICTTGKLPVPWPTLVTTLTYGVQCFSRYPDHMKRHDFFKSAMPEGYVQERTISFKDDGTYKTRAEVKFEGDTLVNRIELKGIDFKEDGNILGHKLEYNFNSHNVYITADKQKNGIKANFKIRHNVEDGSVQLADHYQQNTPIGDGPVLLPDNHYLSTQSVLSKDPNEKRDHMVLLEFVTAAGITHGMDELYK

1. **Strep-sfGFP**

MSAWSHPQFEKSSGSKGEELFTGVVPILVELDGDVNGHKFSVRGEGEGDATNGKLTLKFICTTGKLPVPWPTLVTTLTYGVQCFSRYPDHMKRHDFFKSAMPEGYVQERTISFKDDGTYKTRAEVKFEGDTLVNRIELKGIDFKEDGNILGHKLEYNFNSHNVYITADKQKNGIKANFKIRHNVEDGSVQLADHYQQNTPIGDGPVLLPDNHYLSTQSVLSKDPNEKRDHMVLLEFVTAAGITHGMDELYK

1. **Twin-Strep-sfGFP**

MSAWSHPQFEKGGGSGGGSGGSAWSHPQFEKSSGSKGEELFTGVVPILVELDGDVNGHKFSVRGEGEGDATNGKLTLKFICTTGKLPVPWPTLVTTLTYGVQCFSRYPDHMKRHDFFKSAMPEGYVQERTISFKDDGTYKTRAEVKFEGDTLVNRIELKGIDFKEDGNILGHKLEYNFNSHNVYITADKQKNGIKANFKIRHNVEDGSVQLADHYQQNTPIGDGPVLLPDNHYLSTQSVLSKDPNEKRDHMVLLEFVTAAGITHGMDELYK

1. **6xHis-TEV-sfGFP**

MHHHHHHSSGENLYFQGSSKGEELFTGVVPILVELDGDVNGHKFSVRGEGEGDATNGKLTLKFICTTGKLPVPWPTLVTTLTYGVQCFSRYPDHMKRHDFFKSAMPEGYVQERTISFKDDGTYKTRAEVKFEGDTLVNRIELKGIDFKEDGNILGHKLEYNFNSHNVYITADKQKNGIKANFKIRHNVEDGSVQLADHYQQNTPIGDGPVLLPDNHYLSTQSVLSKDPNEKRDHMVLLEFVTAAGITHGMDELYK

1. **8xHis-TEV-sfGFP**

MHHHHHHHHSSGENLYFQGSSKGEELFTGVVPILVELDGDVNGHKFSVRGEGEGDATNGKLTLKFICTTGKLPVPWPTLVTTLTYGVQCFSRYPDHMKRHDFFKSAMPEGYVQERTISFKDDGTYKTRAEVKFEGDTLVNRIELKGIDFKEDGNILGHKLEYNFNSHNVYITADKQKNGIKANFKIRHNVEDGSVQLADHYQQNTPIGDGPVLLPDNHYLSTQSVLSKDPNEKRDHMVLLEFVTAAGITHGMDELYK

1. **Strep-TEV-sfGFP**

MSAWSHPQFEKSSGENLYFQGSSKGEELFTGVVPILVELDGDVNGHKFSVRGEGEGDATNGKLTLKFICTTGKLPVPWPTLVTTLTYGVQCFSRYPDHMKRHDFFKSAMPEGYVQERTISFKDDGTYKTRAEVKFEGDTLVNRIELKGIDFKEDGNILGHKLEYNFNSHNVYITADKQKNGIKANFKIRHNVEDGSVQLADHYQQNTPIGDGPVLLPDNHYLSTQSVLSKDPNEKRDHMVLLEFVTAAGITHGMDELYK

1. **Twin-Strep-TEV-sfGFP**

MSAWSHPQFEKGGGSGGGSGGSAWSHPQFEKSSGENLYFQGSSKGEELFTGVVPILVELDGDVNGHKFSVRGEGEGDATNGKLTLKFICTTGKLPVPWPTLVTTLTYGVQCFSRYPDHMKRHDFFKSAMPEGYVQERTISFKDDGTYKTRAEVKFEGDTLVNRIELKGIDFKEDGNILGHKLEYNFNSHNVYITADKQKNGIKANFKIRHNVEDGSVQLADHYQQNTPIGDGPVLLPDNHYLSTQSVLSKDPNEKRDHMVLLEFVTAAGITHGMDELYK

1. **6xHis-SUMO-sfGFP**

MGSSHHHHHHSSGLVPRGSHMSDSEVNQEAKPEVKPEVKPETHINLKVSDGSSEIFFKIKKTTPLRRLMEAFAKRQGKEMDSLRFLYDGIRIQADQTPEDLDMEDNDIIEAHREQIGGSGSSKGEELFTGVVPILVELDGDVNGHKFSVRGEGEGDATNGKLTLKFICTTGKLPVPWPTLVTTLTYGVQCFSRYPDHMKRHDFFKSAMPEGYVQERTISFKDDGTYKTRAEVKFEGDTLVNRIELKGIDFKEDGNILGHKLEYNFNSHNVYITADKQKNGIKANFKIRHNVEDGSVQLADHYQQNTPIGDGPVLLPDNHYLSTQSVLSKDPNEKRDHMVLLEFVTAAGITHGMDELYK

1. **8xHis-SUMO-sfGFP**

MGSSHHHHHHHHSSGLVPRGSHMSDSEVNQEAKPEVKPEVKPETHINLKVSDGSSEIFFKIKKTTPLRRLMEAFAKRQGKEMDSLRFLYDGIRIQADQTPEDLDMEDNDIIEAHREQIGGSGSSKGEELFTGVVPILVELDGDVNGHKFSVRGEGEGDATNGKLTLKFICTTGKLPVPWPTLVTTLTYGVQCFSRYPDHMKRHDFFKSAMPEGYVQERTISFKDDGTYKTRAEVKFEGDTLVNRIELKGIDFKEDGNILGHKLEYNFNSHNVYITADKQKNGIKANFKIRHNVEDGSVQLADHYQQNTPIGDGPVLLPDNHYLSTQSVLSKDPNEKRDHMVLLEFVTAAGITHGMDELYK

1. **Strep-SUMO-sfGFP**

MGSSSAWSHPQFEKSSGLVPRGSHMSDSEVNQEAKPEVKPEVKPETHINLKVSDGSSEIFFKIKKTTPLRRLMEAFAKRQGKEMDSLRFLYDGIRIQADQTPEDLDMEDNDIIEAHREQIGGSGSSKGEELFTGVVPILVELDGDVNGHKFSVRGEGEGDATNGKLTLKFICTTGKLPVPWPTLVTTLTYGVQCFSRYPDHMKRHDFFKSAMPEGYVQERTISFKDDGTYKTRAEVKFEGDTLVNRIELKGIDFKEDGNILGHKLEYNFNSHNVYITADKQKNGIKANFKIRHNVEDGSVQLADHYQQNTPIGDGPVLLPDNHYLSTQSVLSKDPNEKRDHMVLLEFVTAAGITHGMDELYK

1. **Twin-Strep-SUMO-sfGFP**

MGSSSAWSHPQFEKGGGSGGGSGGSAWSHPQFEKSSGLVPRGSHMSDSEVNQEAKPEVKPEVKPETHINLKVSDGSSEIFFKIKKTTPLRRLMEAFAKRQGKEMDSLRFLYDGIRIQADQTPEDLDMEDNDIIEAHREQIGGSGSSKGEELFTGVVPILVELDGDVNGHKFSVRGEGEGDATNGKLTLKFICTTGKLPVPWPTLVTTLTYGVQCFSRYPDHMKRHDFFKSAMPEGYVQERTISFKDDGTYKTRAEVKFEGDTLVNRIELKGIDFKEDGNILGHKLEYNFNSHNVYITADKQKNGIKANFKIRHNVEDGSVQLADHYQQNTPIGDGPVLLPDNHYLSTQSVLSKDPNEKRDHMVLLEFVTAAGITHGMDELYK

1. **FLAG-sfGFP**

MDYKDDDDKSSGSKGEELFTGVVPILVELDGDVNGHKFSVRGEGEGDATNGKLTLKFICTTGKLPVPWPTLVTTLTYGVQCFSRYPDHMKRHDFFKSAMPEGYVQERTISFKDDGTYKTRAEVKFEGDTLVNRIELKGIDFKEDGNILGHKLEYNFNSHNVYITADKQKNGIKANFKIRHNVEDGSVQLADHYQQNTPIGDGPVLLPDNHYLSTQSVLSKDPNEKRDHMVLLEFVTAAGITHGMDELYK

1. **3xFLAG-sfGFP**

MDYKDHDGDYKDHDIDYKDDDDKSSGSKGEELFTGVVPILVELDGDVNGHKFSVRGEGEGDATNGKLTLKFICTTGKLPVPWPTLVTTLTYGVQCFSRYPDHMKRHDFFKSAMPEGYVQERTISFKDDGTYKTRAEVKFEGDTLVNRIELKGIDFKEDGNILGHKLEYNFNSHNVYITADKQKNGIKANFKIRHNVEDGSVQLADHYQQNTPIGDGPVLLPDNHYLSTQSVLSKDPNEKRDHMVLLEFVTAAGITHGMDELYK

1. **sfGFP-6xHis**

MSKGEELFTGVVPILVELDGDVNGHKFSVRGEGEGDATNGKLTLKFICTTGKLPVPWPTLVTTLTYGVQCFSRYPDHMKRHDFFKSAMPEGYVQERTISFKDDGTYKTRAEVKFEGDTLVNRIELKGIDFKEDGNILGHKLEYNFNSHNVYITADKQKNGIKANFKIRHNVEDGSVQLADHYQQNTPIGDGPVLLPDNHYLSTQSVLSKDPNEKRDHMVLLEFVTAAGITHGMDELYKSSGHHHHHH

1. **sfGFP-8xHis**

MSKGEELFTGVVPILVELDGDVNGHKFSVRGEGEGDATNGKLTLKFICTTGKLPVPWPTLVTTLTYGVQCFSRYPDHMKRHDFFKSAMPEGYVQERTISFKDDGTYKTRAEVKFEGDTLVNRIELKGIDFKEDGNILGHKLEYNFNSHNVYITADKQKNGIKANFKIRHNVEDGSVQLADHYQQNTPIGDGPVLLPDNHYLSTQSVLSKDPNEKRDHMVLLEFVTAAGITHGMDELYKSSGHHHHHHHH

1. **sfGFP-strep**

MSKGEELFTGVVPILVELDGDVNGHKFSVRGEGEGDATNGKLTLKFICTTGKLPVPWPTLVTTLTYGVQCFSRYPDHMKRHDFFKSAMPEGYVQERTISFKDDGTYKTRAEVKFEGDTLVNRIELKGIDFKEDGNILGHKLEYNFNSHNVYITADKQKNGIKANFKIRHNVEDGSVQLADHYQQNTPIGDGPVLLPDNHYLSTQSVLSKDPNEKRDHMVLLEFVTAAGITHGMDELYKSSGSAWSHPQFEK

1. **sfGFP-Twin-Strep**

MSKGEELFTGVVPILVELDGDVNGHKFSVRGEGEGDATNGKLTLKFICTTGKLPVPWPTLVTTLTYGVQCFSRYPDHMKRHDFFKSAMPEGYVQERTISFKDDGTYKTRAEVKFEGDTLVNRIELKGIDFKEDGNILGHKLEYNFNSHNVYITADKQKNGIKANFKIRHNVEDGSVQLADHYQQNTPIGDGPVLLPDNHYLSTQSVLSKDPNEKRDHMVLLEFVTAAGITHGMDELYKSSGSAWSHPQFEKGGGSGGGSGGSAWSHPQFEK

1. **sfGFP-C**

MSKGEELFTGVVPILVELDGDVNGHKFSVRGEGEGDATNGKLTLKFICTTGKLPVPWPTLVTTLTYGVQCFSRYPDHMKRHDFFKSAMPEGYVQERTISFKDDGTYKTRAEVKFEGDTLVNRIELKGIDFKEDGNILGHKLEYNFNSHNVYITADKQKNGIKANFKIRHNVEDGSVQLADHYQQNTPIGDGPVLLPDNHYLSTQSVLSKDPNEKRDHMVLLEFVTAAGITHGMDELYKSSGEPEA

1. **sfGFP-TEV-6xHis**

MSKGEELFTGVVPILVELDGDVNGHKFSVRGEGEGDATNGKLTLKFICTTGKLPVPWPTLVTTLTYGVQCFSRYPDHMKRHDFFKSAMPEGYVQERTISFKDDGTYKTRAEVKFEGDTLVNRIELKGIDFKEDGNILGHKLEYNFNSHNVYITADKQKNGIKANFKIRHNVEDGSVQLADHYQQNTPIGDGPVLLPDNHYLSTQSVLSKDPNEKRDHMVLLEFVTAAGITHGMDELYKSENLYFQGSSGHHHHHH

1. **sfGFP-TEV-8xHis**

MSKGEELFTGVVPILVELDGDVNGHKFSVRGEGEGDATNGKLTLKFICTTGKLPVPWPTLVTTLTYGVQCFSRYPDHMKRHDFFKSAMPEGYVQERTISFKDDGTYKTRAEVKFEGDTLVNRIELKGIDFKEDGNILGHKLEYNFNSHNVYITADKQKNGIKANFKIRHNVEDGSVQLADHYQQNTPIGDGPVLLPDNHYLSTQSVLSKDPNEKRDHMVLLEFVTAAGITHGMDELYKSENLYFQGSSGHHHHHHHH

1. **sfGFP-TEV-strep**

MSKGEELFTGVVPILVELDGDVNGHKFSVRGEGEGDATNGKLTLKFICTTGKLPVPWPTLVTTLTYGVQCFSRYPDHMKRHDFFKSAMPEGYVQERTISFKDDGTYKTRAEVKFEGDTLVNRIELKGIDFKEDGNILGHKLEYNFNSHNVYITADKQKNGIKANFKIRHNVEDGSVQLADHYQQNTPIGDGPVLLPDNHYLSTQSVLSKDPNEKRDHMVLLEFVTAAGITHGMDELYKSENLYFQGSSGSAWSHPQFEK

1. **sfGFP-TEV-Twin-Strep**

MSKGEELFTGVVPILVELDGDVNGHKFSVRGEGEGDATNGKLTLKFICTTGKLPVPWPTLVTTLTYGVQCFSRYPDHMKRHDFFKSAMPEGYVQERTISFKDDGTYKTRAEVKFEGDTLVNRIELKGIDFKEDGNILGHKLEYNFNSHNVYITADKQKNGIKANFKIRHNVEDGSVQLADHYQQNTPIGDGPVLLPDNHYLSTQSVLSKDPNEKRDHMVLLEFVTAAGITHGMDELYKSENLYFQGSSGSAWSHPQFEKGGGSGGGSGGSAWSHPQFEK

1. **sfGFP-TEV-C**

MSKGEELFTGVVPILVELDGDVNGHKFSVRGEGEGDATNGKLTLKFICTTGKLPVPWPTLVTTLTYGVQCFSRYPDHMKRHDFFKSAMPEGYVQERTISFKDDGTYKTRAEVKFEGDTLVNRIELKGIDFKEDGNILGHKLEYNFNSHNVYITADKQKNGIKANFKIRHNVEDGSVQLADHYQQNTPIGDGPVLLPDNHYLSTQSVLSKDPNEKRDHMVLLEFVTAAGITHGMDELYKSENLYFQGSSGEPEA

1. **sfGFP-TEV-FLAG**

MSKGEELFTGVVPILVELDGDVNGHKFSVRGEGEGDATNGKLTLKFICTTGKLPVPWPTLVTTLTYGVQCFSRYPDHMKRHDFFKSAMPEGYVQERTISFKDDGTYKTRAEVKFEGDTLVNRIELKGIDFKEDGNILGHKLEYNFNSHNVYITADKQKNGIKANFKIRHNVEDGSVQLADHYQQNTPIGDGPVLLPDNHYLSTQSVLSKDPNEKRDHMVLLEFVTAAGITHGMDELYKSSGDYKDDDDK

1. **sfGFP-TEV-3xFLAG**

MSKGEELFTGVVPILVELDGDVNGHKFSVRGEGEGDATNGKLTLKFICTTGKLPVPWPTLVTTLTYGVQCFSRYPDHMKRHDFFKSAMPEGYVQERTISFKDDGTYKTRAEVKFEGDTLVNRIELKGIDFKEDGNILGHKLEYNFNSHNVYITADKQKNGIKANFKIRHNVEDGSVQLADHYQQNTPIGDGPVLLPDNHYLSTQSVLSKDPNEKRDHMVLLEFVTAAGITHGMDELYKSSGDYKDHDGDYKDHDIDYKDDDDK

1. **8xHis-mCherry**

MHHHHHHHHSSGVSKGEEDNMAIIKEFMRFKVHMEGSVNGHEFEIEGEGEGRPYEGTQTAKLKVTKGGPLPFAWDILSPQFMYGSKAYVKHPADIPDYLKLSFPEGFKWERVMNFEDGGVVTVTQDSSLQDGEFIYKVKLRGTNFPSDGPVMQKKTMGWEASSERMYPEDGALKGEIKQRLKLKDGGHYDAEVKTTYKAKKPVQLPGAYNVNIKLDITSHNEDYTIVEQYERAEGRHSTGGMDELYK

1. **mCherry-8xHis**

MVSKGEEDNMAIIKEFMRFKVHMEGSVNGHEFEIEGEGEGRPYEGTQTAKLKVTKGGPLPFAWDILSPQFMYGSKAYVKHPADIPDYLKLSFPEGFKWERVMNFEDGGVVTVTQDSSLQDGEFIYKVKLRGTNFPSDGPVMQKKTMGWEASSERMYPEDGALKGEIKQRLKLKDGGHYDAEVKTTYKAKKPVQLPGAYNVNIKLDITSHNEDYTIVEQYERAEGRHSTGGMDELYKSSGHHHHHHHH

1. **Strep-mCherry**

MSAWSHPQFEKSSGVSKGEEDNMAIIKEFMRFKVHMEGSVNGHEFEIEGEGEGRPYEGTQTAKLKVTKGGPLPFAWDILSPQFMYGSKAYVKHPADIPDYLKLSFPEGFKWERVMNFEDGGVVTVTQDSSLQDGEFIYKVKLRGTNFPSDGPVMQKKTMGWEASSERMYPEDGALKGEIKQRLKLKDGGHYDAEVKTTYKAKKPVQLPGAYNVNIKLDITSHNEDYTIVEQYERAEGRHSTGGMDELYK

1. **mCherry-Strep**

MVSKGEEDNMAIIKEFMRFKVHMEGSVNGHEFEIEGEGEGRPYEGTQTAKLKVTKGGPLPFAWDILSPQFMYGSKAYVKHPADIPDYLKLSFPEGFKWERVMNFEDGGVVTVTQDSSLQDGEFIYKVKLRGTNFPSDGPVMQKKTMGWEASSERMYPEDGALKGEIKQRLKLKDGGHYDAEVKTTYKAKKPVQLPGAYNVNIKLDITSHNEDYTIVEQYERAEGRHSTGGMDELYKSSGSAWSHPQFEK

1. **8xHis-ADH**

MHHHHHHHHSSGMEFRHNLPSSDIIFGSGTLEKIGEETKKWGDKAILVTGKSNMKKLGFLADAIDYLESAGVETVHYGEIEPNPTTTVVDEGAEIVLEEGCDVVVALGGGSSMDAAKGIAMVAGHSAEERDISVWDFAPEGDKETKPITEKTLPVIAATSTSGTGSHVTPYAVITNPETKGKPGFGNKHSFPKVSIVDIDILKEMPPRLTAITGYDVFSHVSENLTAKGDHPTADPLAIRAIEYVTEYLLRAVEDGEDIKAREKMAVADTYAGLSNTISGTTLRHAMAHPISGYYPDISHGQALASISVPIMEHNIENGDEKTWERYSRIAVALDASKPVDNTRQAASKAVDGLKNLLRSLDLDKPLSELGVEEEKIPEMTEGAFIYMGGGIEANPVDVSKEDVKEIFRKSL

1. **ADH-8xHis**

MEFRHNLPSSDIIFGSGTLEKIGEETKKWGDKAILVTGKSNMKKLGFLADAIDYLESAGVETVHYGEIEPNPTTTVVDEGAEIVLEEGCDVVVALGGGSSMDAAKGIAMVAGHSAEERDISVWDFAPEGDKETKPITEKTLPVIAATSTSGTGSHVTPYAVITNPETKGKPGFGNKHSFPKVSIVDIDILKEMPPRLTAITGYDVFSHVSENLTAKGDHPTADPLAIRAIEYVTEYLLRAVEDGEDIKAREKMAVADTYAGLSNTISGTTLRHAMAHPISGYYPDISHGQALASISVPIMEHNIENGDEKTWERYSRIAVALDASKPVDNTRQAASKAVDGLKNLLRSLDLDKPLSELGVEEEKIPEMTEGAFIYMGGGIEANPVDVSKEDVKEIFRKSLSSGHHHHHHHH

1. **Strep-ADH**

MSAWSHPQFEKSSGMEFRHNLPSSDIIFGSGTLEKIGEETKKWGDKAILVTGKSNMKKLGFLADAIDYLESAGVETVHYGEIEPNPTTTVVDEGAEIVLEEGCDVVVALGGGSSMDAAKGIAMVAGHSAEERDISVWDFAPEGDKETKPITEKTLPVIAATSTSGTGSHVTPYAVITNPETKGKPGFGNKHSFPKVSIVDIDILKEMPPRLTAITGYDVFSHVSENLTAKGDHPTADPLAIRAIEYVTEYLLRAVEDGEDIKAREKMAVADTYAGLSNTISGTTLRHAMAHPISGYYPDISHGQALASISVPIMEHNIENGDEKTWERYSRIAVALDASKPVDNTRQAASKAVDGLKNLLRSLDLDKPLSELGVEEEKIPEMTEGAFIYMGGGIEANPVDVSKEDVKEIFRKSL

1. **ADH-Strep**

MEFRHNLPSSDIIFGSGTLEKIGEETKKWGDKAILVTGKSNMKKLGFLADAIDYLESAGVETVHYGEIEPNPTTTVVDEGAEIVLEEGCDVVVALGGGSSMDAAKGIAMVAGHSAEERDISVWDFAPEGDKETKPITEKTLPVIAATSTSGTGSHVTPYAVITNPETKGKPGFGNKHSFPKVSIVDIDILKEMPPRLTAITGYDVFSHVSENLTAKGDHPTADPLAIRAIEYVTEYLLRAVEDGEDIKAREKMAVADTYAGLSNTISGTTLRHAMAHPISGYYPDISHGQALASISVPIMEHNIENGDEKTWERYSRIAVALDASKPVDNTRQAASKAVDGLKNLLRSLDLDKPLSELGVEEEKIPEMTEGAFIYMGGGIEANPVDVSKEDVKEIFRKSLSSGSAWSHPQFEK

1. **8xHis-Twin-Strep-sfGFP**

MHHHHHHHHGTSGWSHPQFEKGGSGWSHPQFEKSSGSKGEELFTGVVPILVELDGDVNGHKFSVRGEGEGDATNGKLTLKFICTTGKLPVPWPTLVTTLTYGVQCFSRYPDHMKRHDFFKSAMPEGYVQERTISFKDDGTYKTRAEVKFEGDTLVNRIELKGIDFKEDGNILGHKLEYNFNSHNVYITADKQKNGIKANFKIRHNVEDGSVQLADHYQQNTPIGDGPVLLPDNHYLSTQSVLSKDPNEKRDHMVLLEFVTAAGITHGMDELYK

1. **8xHis-TwinS-trep-mCherry**

MHHHHHHHHGTSGWSHPQFEKGGSGWSHPQFEKSSGVSKGEEDNMAIIKEFMRFKVHMEGSVNGHEFEIEGEGEGRPYEGTQTAKLKVTKGGPLPFAWDILSPQFMYGSKAYVKHPADIPDYLKLSFPEGFKWERVMNFEDGGVVTVTQDSSLQDGEFIYKVKLRGTNFPSDGPVMQKKTMGWEASSERMYPEDGALKGEIKQRLKLKDGGHYDAEVKTTYKAKKPVQLPGAYNVNIKLDITSHNEDYTIVEQYERAEGRHSTGGMDELYK

1. **8xHis-Twin-Strep-ADH**

MHHHHHHHHGTSGWSHPQFEKGGSGWSHPQFEKSSGMEFRHNLPSSDIIFGSGTLEKIGEETKKWGDKAILVTGKSNMKKLGFLADAIDYLESAGVETVHYGEIEPNPTTTVVDEGAEIVLEEGCDVVVALGGGSSMDAAKGIAMVAGHSAEERDISVWDFAPEGDKETKPITEKTLPVIAATSTSGTGSHVTPYAVITNPETKGKPGFGNKHSFPKVSIVDIDILKEMPPRLTAITGYDVFSHVSENLTAKGDHPTADPLAIRAIEYVTEYLLRAVEDGEDIKAREKMAVADTYAGLSNTISGTTLRHAMAHPISGYYPDISHGQALASISVPIMEHNIENGDEKTWERYSRIAVALDASKPVDNTRQAASKAVDGLKNLLRSLDLDKPLSELGVEEEKIPEMTEGAFIYMGGGIEANPVDVSKEDVKEIFRKSL

**Figure S5.** Tryptic digest and LC-MS/MS analysis. Tryptic digest and LC-MS/MS analysis of all the sfGFP, mCherry, and halophilic alcohol dehydrogenase (ADH) constructs with purification tags used in this study. Matched peptides are underlined*.*

**Figure S6.** SDS-PAGE analysis of the purification of the sfGFP with C-tag. (left) C-tag sfGFP constructs with TEV cleavage site (right). Lane M: molecular weight marker; Lane 1: Elution from C-tag XL affinity column using 2 M MgCl_2_. The column was washed with phosphate buffer pH 7.4 containing 1 M NaCl before elution.

sfGFP

AGCAAGGGCGAGGAGCTCTTCACCGGCGTCGTCCCCATCCTCGTCGAGCTCGACGGCGACGTCAACGGCCACAAGTTCAGCGTCCGCGGCGAGGGCGAGGGCGACGCCACCAACGGCAAGCTCACCCTCAAGTTCATCTGCACCACCGGCAAGCTCCCCGTCCCCTGGCCCACCCTCGTCACCACCCTCACCTACGGCGTCCAGTGCTTCAGCCGCTACCCCGACCACATGAAGCGCCACGACTTCTTCAAGAGCGCCATGCCCGAGGGCTACGTCCAGGAGCGCACCATCAGCTTCAAGGACGACGGCACCTACAAGACCCGCGCCGAGGTCAAGTTCGAGGGCGACACCCTCGTCAACCGCATCGAGCTCAAGGGCATCGACTTCAAGGAGGACGGCAACATCCTCGGCCACAAGCTCGAGTACAACTTCAACAGCCACAACGTCTACATCACCGCCGACAAGCAGAAGAACGGCATCAAGGCCAACTTCAAGATCCGCCACAACGTCGAGGACGGCAGCGTCCAGCTCGCCGACCACTACCAGCAGAACACCCCCATCGGCGACGGCCCCGTCCTCCTCCCCGACAACCACTACCTCAGCACCCAGAGCGTCCTCAGCAAGGACCCCAACGAGAAGCGCGACCACATGGTCCTCCTCGAGTTCGTCACCGCCGCCGGCATCACCCACGGCATGGACGAGCTCTACAAGTGA

mCherry

GTCAGCAAGGGCGAGGAGGACAACATGGCCATCATCAAGGAGTTCATGCGCTTCAAGGTCCACATGGAGGGCAGCGTCAACGGCCACGAGTTCGAGATCGAGGGCGAGGGCGAGGGCCGCCCCTACGAGGGCACCCAGACCGCCAAGCTCAAGGTCACCAAGGGCGGCCCCCTCCCCTTCGCCTGGGACATCCTCAGCCCCCAGTTCATGTACGGCAGCAAGGCCTACGTCAAGCACCCCGCCGACATCCCCGACTACCTCAAGCTCAGCTTCCCCGAGGGCTTCAAGTGGGAGCGCGTCATGAACTTCGAGGACGGCGGCGTCGTCACCGTCACCCAGGACAGCAGCCTCCAGGACGGCGAGTTCATCTACAAGGTCAAGCTCCGCGGCACCAACTTCCCCAGCGACGGCCCCGTCATGCAGAAGAAGACCATGGGCTGGGAGGCCAGCAGCGAGCGCATGTACCCCGAGGACGGCGCCCTCAAGGGCGAGATCAAGCAGCGCCTCAAGCTCAAGGACGGCGGCCACTACGACGCCGAGGTCAAGACCACCTACAAGGCCAAGAAGCCCGTCCAGCTCCCCGGCGCCTACAACGTCAACATCAAGCTCGACATCACCAGCCACAACGAGGACTACACCATCGTCGAGCAGTACGAGCGCGCCGAGGGCCGCCACAGCACCGGCGGCATGGACGAGCTCTACAAG

ADH

ATGGAGTTCCGCCACAACCTCCCCAGCAGCGACATCATCTTCGGCAGCGGCACCCTCGAGAAGATCGGCGAGGAGACCAAGAAGTGGGGCGACAAGGCCATCCTCGTCACCGGCAAGAGCAACATGAAGAAGCTCGGCTTCCTCGCCGACGCCATCGACTACCTCGAGAGCGCCGGCGTCGAGACCGTCCACTACGGCGAGATCGAGCCCAACCCCACCACCACCGTCGTCGACGAGGGCGCCGAGATCGTCCTCGAGGAGGGCTGCGACGTCGTCGTCGCCCTCGGCGGCGGCAGCAGCATGGACGCCGCCAAGGGCATCGCCATGGTCGCCGGCCACAGCGCCGAGGAGCGCGACATCAGCGTCTGGGACTTCGCCCCCGAGGGCGACAAGGAGACCAAGCCCATCACCGAGAAGACCCTCCCCGTCATCGCCGCCACCAGCACCAGCGGCACCGGCAGCCACGTCACCCCCTACGCCGTCATCACCAACCCCGAGACCAAGGGCAAGCCCGGCTTCGGCAACAAGCACAGCTTCCCCAAGGTCAGCATCGTCGACATCGACATCCTCAAGGAGATGCCCCCCCGCCTCACCGCCATCACCGGCTACGACGTCTTCAGCCACGTCAGCGAGAACCTCACCGCCAAGGGCGACCACCCCACCGCCGACCCCCTCGCCATCCGCGCCATCGAGTACGTCACCGAGTACCTCCTCCGCGCCGTCGAGGACGGCGAGGACATCAAGGCCCGCGAGAAGATGGCCGTCGCCGACACCTACGCCGGCCTCAGCAACACCATCAGCGGCACCACCCTCCGCCACGCCATGGCCCACCCCATCAGCGGCTACTACCCCGACATCAGCCACGGCCAGGCCCTCGCCAGCATCAGCGTCCCCATCATGGAGCACAACATCGAGAACGGCGACGAGAAGACCTGGGAGCGCTACAGCCGCATCGCCGTCGCCCTCGACGCCAGCAAGCCCGTCGACAACACCCGCCAGGCCGCCAGCAAGGCCGTCGACGGCCTCAAGAACCTCCTCCGCAGCCTCGACCTCGACAAGCCCCTCAGCGAGCTCGGCGTCGAGGAGGAGAAGATCCCCGAGATGACCGAGGGCGCCTTCATCTACATGGGCGGCGGCATCGAGGCCAACCCCGTCGACGTCAGCAAGGAGGACGTCAAGGAGATCTTCCGCAAGAGCCTC

**Figure S7.** Nucleotide sequences of sfGFP, mCherry and ADH.
